# Correction: Determining cost and placement decisions for moderate complexity NAATs for tuberculosis drug susceptibility testing

**DOI:** 10.1371/journal.pone.0316653

**Published:** 2024-12-26

**Authors:** Akash Malhotra, Ryan Thompson, Margaretha De Vos, Anura David, Samuel Schumacher, Hojoon Sohn

The affiliation for the fifth author is incorrect. Samuel Schumacher is not affiliated with #4 but with #2: Foundation for Innovative New Diagnostics (FIND), Geneva, Switzerland.
