# Supplementary material for: Determining cost and placement decisions for moderate complexity NAATs for tuberculosis drug susceptibility testing
Source: PLoS One. 2023 Aug 24;18(8):e0290496. doi: 10.1371/journal.pone.0290496 (PMC10449112; doi:10.1371/journal.pone.0290496)
Supplement: S1 Table — For each discrete activity, two separate time estimates were measured: direct hands-on time of laboratory personnel performing the test and full procedural step to account for laboratory instrument use. As we were not able to measure activity times for various sample batch sizes, we estimated times required to process lower (12 or 24 samples per batch) or higher (96 samples per batch) batch sizes based on multiple measurement (at least three) of typical batch size processed during the FIND’s external laboratory evaluation study. In estimating times required to process lower or higher batch sizes (vs. observed), we first calculated per-sample processing times for testing steps for which procedural and hands-on time could vary based on the batch size and calculated estimated total time for each step. (DOCX) [file pone.0290496.s001.docx]

**S1 Table: Mean/median duration time per procedure for Low Throughput NAAT and High Throughput NAAT.**

|  |  | Low Throughput NAAT | | High Throughput NAAT | | |
| --- | --- | --- | --- | --- | --- | --- |
| Procedure | Time designation | *Direct* | *Estimated* | *Estimated* | *Direct* | *Estimated* |
|  |  | 24 | 12 | 96 | 52 | 24 |
|  |  | *Max* | *Low* | *Max* | *Med* | *Low* |
| Instrument setup | STAFF TIME | 5.7 | 5.7 | 49.47 | 49.47 | 49.47 |
| Reagent reconstitution |  | 0 | 0 | 19.6 | 19.6 | 19.6 |
| Inactivation (addition of buffer) |  | 4.6 | 3.4 | 28.2 | 14.1 | 10.6 |
| Incubation |  | 30 | 30 | 30 | 30 | 30 |
| Load instrument |  | 6.1 | 4.6 | 11.7 | 5.9 | 4.4 |
| Total run time | PROCEDURE  TIME | 221 | 180 | 222.9 | 181.9 | 149.9 |
| Instrument run time (DNA Extraction) |  | 130 | 89 | 121 | 80 | 48 |
| Sealing plates/tubes |  | 0 | 0 | 0.9 | 0.9 | 0.9 |
| Instrument run time (PCR amplification/detection) |  | 91 | 91 | 101 | 101 | 101 |
| Unload instrument | STAFF TIME | 4.8 | 2.4 | 13.1 | 6.5 | 3.3 |
| Post analytic process |  | 4.6 | 4.6 | 2.2 | 2.2 | 2.2 |
|  |  |  |  |  |  |  |
| TOTAL TIME | Staff + Procedure | 497.8 | 410.7 | 599.9 | 491.5 | 419.2 |
| DIRECT STAFF TIME | Staff Only | 55.8 | 50.7 | 154.2 | 127.7 | 119.4 |
