# Supplementary figures and images for: Determining cost and placement decisions for moderate complexity NAATs for tuberculosis drug susceptibility testing
Source: PLoS One. 2023 Aug 24;18(8):e0290496. doi: 10.1371/journal.pone.0290496 (PMC10449112; doi:10.1371/journal.pone.0290496)

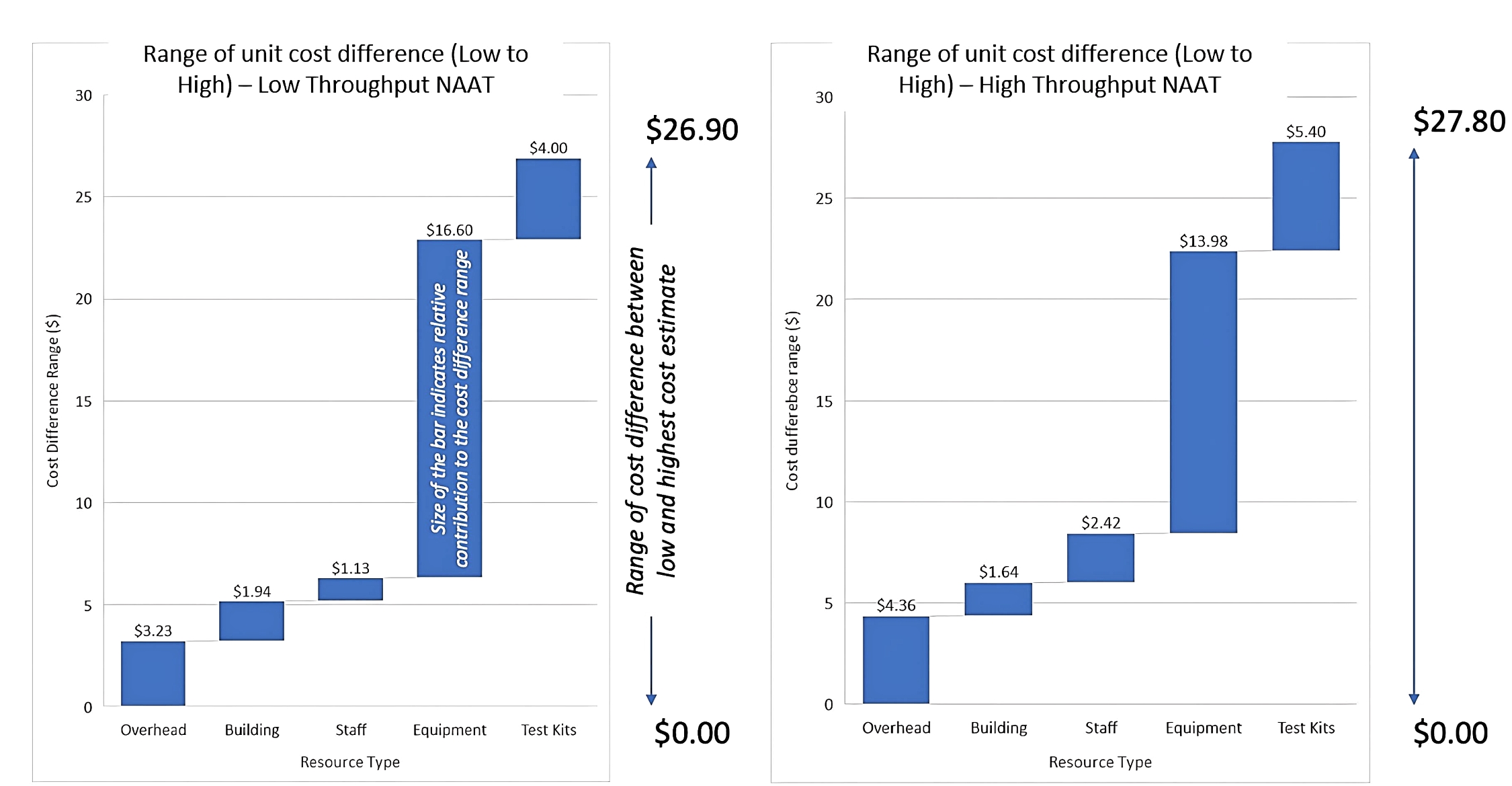

Supplement: S1 Fig — The size of the bar indicates relative contribution of a cost component to the cost difference range. (JPG) [file pone.0290496.s002.jpg]
